# Luteal activity of Abadeh ecotype does in summer and winter and the effect of kisspeptin-10 on luteinizing hormone secretion in the anestrus does

**Published:** 2014

**Authors:** Mohammad Arjmand, Abdolah Mirzaei, Mohammad Reza Jafarzadeh Shirazi, Amin Tamadon, Mohammad Saied Salehi, Mehdi Saeb, Mohammad Reza Namavar, Mohammad Reza Zandi, Hojatollah Shahheidari, Sara Moradi

**Affiliations:** 1 *Department of Animal Science, College of Agriculture, Shiraz University, Shiraz, Iran; *; 2 * Department of Clinical Sciences, School of Veterinary Medicine, Shiraz University, Shiraz, Iran; *; 3 * Transgenic Technology Research Center, Shiraz University of Medical Sciences, Shiraz, Iran; *; 4 *Department of Basic Sciences, School of Veterinary Medicine, Shiraz University, Shiraz, Iran; *; 5 * Histomorphometry and Stereology Research Center, Shiraz University of Medical Sciences and Department of Anatomical Sciences, School of Medicine, Shiraz University of Medical Sciences, Shiraz, Iran.*

**Keywords:** Anestrus, Goat, Kisspeptin, Luteal activity, Luteinizing hormone

## Abstract

The aims of the present study were to evaluate luteal activity in Abadeh ecotype goat during summer and winter and also the effect of a single dose kisspeptin-10 injection on the secretion of luteinizing hormone (LH) in female anestrous goats. In the first study, progesterone (P_4_) concentration in 10 goats in summer (n = 6) and winter (n = 4) were measured every other day. Moreover, in summer group, a male teaser goat was left in the herd on days of sampling for one hour. Goats with P_4_ concentration ≥1 ng mL^-1^, at least two consecutive measurements, were considered with luteal activity. In the second study, the anestrous phase was confirmed by P_4_ measurement 20 and 10 days before the kisspeptin injection in five female Abadeh ecotype goats (4 to 5 years old). The goats were given a single IV injection of saline (2 mL) as control group and the same goats (1 hr after the last blood sampling) were given kisspeptin (1 μg kg^-1^) as treatment group. The blood samples were collected at –60, –40, –20 and 0 min (before injection), and 10, 20, 30, 40, 50, 60, 80, 100, 120 and 140 min after the injection and LH concentration were measured. A single IV injection of 1 µg kg^-1^ of kisspeptin-10 did not stimulate the release of LH in female anestrous goats. In summer, in the presence of teaser goat, luteal activity was seen in all goats. In the absence of male goat in winter, some goats showed luteal activity and others showed anestrus.

## Introduction

Goat farming plays an important role in the agricultural economy of Fars province, Iran. Based on the annual report of the Veterinary Organization of Fars province, about 6.3 million goats exist in Fars province and most of them are kept by nomads.^   1 ^ In intensive goat production for easier management of gestation, parturition and kids husbandry, does were kept without bucks and in breeding season bucks were introduced to the herd; therefore, determination of the months that reproduction axis become activated is important.^   2 ^ In general, goat is a short day seasonal breeder ruminant with 21 days estrous cycle and its breeding season starts in early autumn and finished in late winter.^   3 ^ However, unpublished observations in local herds and slaughterhouses show that Fars native goats have different patterns in breeding season and reproductive performance that reflect their ovarian activity. 

Pulsatile secretion of gonadotropin releasing hormone (GnRH) from the hypothalamus regulates the hypo-thalamo-pituitary gonadal (HPG) axis. During breeding season, high level of progesterone (P_4_) secreted from corpus luteum inhibits GnRH/luteinizing hormone (LH) pulse frequency. Thus, when corpus luteum regressed, GnRH/LH pulse frequencies increase and LH stimulate estradiol secretion. High level of estradiol leads to LH surge and ovulation. On the other hand, during the non-breeding season, ovaries become inactive and P_4_ secretion maintain at the basal level. Previous researches reported that basal level of P_4_ in Abadeh, Markhoz and Creole goats is 0.5, 1, and 1 ng mL^-1^, respectively.^4^^-^^6^ The first aim of study was to investigate luteal activity based on serum P_4_ concentration in Abadeh ecotype goats during the summer (transitional period from anoestrus to the natural breeding season) and winter (transitional period from the natural breeding season to anoestrus).

The integration of a series of central neuronal inputs that mediate environmental influences as well as sex steroid feedback and other endogenous factors (metabolic signals, stress hormones, and etc.) involve in the reproductive regulation.^7^^,^^8^ Male presentation in anestrous season to females in goats group induced LH secretion^   9 ^ and increased the multiple-unit activity (MUA)^   10 ^ within the arcuate nucleus (ARC) thought to represent a GnRH pulse modulator.^   11 ^ Moreover, the MUA recordings were noted to be in close proximity to kisspeptin neurons.^12^^,^^13^ Kisspeptin, the product of the KiSS-1 gene, encodes a 145-amino acid peptide that is further processed to generate biologically active peptides of various lengths (10–54 amino acids) termed kisspeptins.^   14 ^ Kisspeptin neurons are found in the hypothalamus, and secretion of kisspeptin strongly stimulates the secretion of gonadotropin through a G protein-coupled receptor known as GPR54.^   14 ^ The actions of these peptides are thought to involve in stimulating GnRH neuronal activity through GPR54, although the possibility of additional sites of action (e.g. at the pituitary) cannot be ruled out.^   14 ^ Kisspeptin stimulates LH secretion in a GnRH dependant manner by increasing GnRH secretion into the hypophysial portal blood.^   15 ^^,^^   16 ^ There are several studies in different mammalians species with different doses, routes of administration, sexes and types of kisspeptin peptide for induction of the LH release which can have effects on levels of LH (Table 1).

In goat, kisspeptin neurons are identified in the ARC during the follicular phase, luteal phase and anestrous stage.^   17 ^ Kiss-1 expression is markedly up-regulated in the ARC at the onset of the breeding season.^3^^,^^18^ In addition, the number of kisspeptin neurons in goat is higher in the breeding season than non-breeding season.^   3 ^ An administration of kisspeptin, either centrally or peripherally, was shown to elicit the release of gonadotropin in ruminants^19^^-^^23^ as well as in rats,^   24 ^ pigs,^   25 ^ monkeys^   26 ^ and humans.^27^^,^^28^ In ruminants, kisspeptin has been examined mainly in sheep,^18^^-^^21^^,^^29^^,^^30^ with a few studies also conducted in cattle.^22^^,^^23^^,^^31^^,^^32^ However, there is a hypothesis that suggests kisspeptin may stimulate the release of gonadotropin in anestrous goats. Therefore, the second aim of study was to investigate the effect of a single dose injection of kisspeptin-10 on the release of LH in vivo in female anestrous goats.

## Materials and Methods

All experimental protocols were approved by the Committee for Animal Experiments of Shiraz University. Both studies were conducted in the Research Station, College of Agriculture, Shiraz University (latitude of 29˚ 44' N and longitude of 52˚ 37' E, 1810 m above sea level). The goats were housed in open shed stalls and fed alfalfa hay, wheat straw and concentrate according to NRC;^33^ water was available *ad libitum*. One month before the start of the experiments, anti-parasitic treatment with ivermectin was conducted.

**Study 1: Reproductive activity of Abadeh ecotype goat. **Ten non-pregnant, non-lactating Abadeh ecotype multiparous female goats, (4 to 5 year-old and mean weight 40 kg) kept without the presence of male goats were used. Experimental periods were from 7 to 29 August 2011 in summer (mean temperature = 27.7 ˚C, day length = 13.1 ± 0.1 hr, n = 6), and in winter from 1 to 17 January 2012 (mean temperature = 7.3 ˚C, day length = 10.4 ± 0.1 hr, n = 4), respectively. In both seasons, blood samples were collected from all goats through the jugular vein to measure P_4_ thrice weekly. Samples were allowed to clot at room temperature for up to 1 hr, centrifuged for serum harvest (for 10 min at 3000 *g*) and serum samples were stored at –22 ˚C until assayed. Serum P_4_ concentration were determined using a validated commercial radio-immunoassay kit (Immunotech kit, Marseille, France). The intra- and inter-assay coefficients of variation (CVs) of the assays were 5.8% and 9.0%, respectively. The sensitivity of the test was 0.05 ng/mL, and the recovery rate of the assay ranged from 85.0% to 110%. Furthermore, to detect estrus during the days of sampling in non-breeding season (summer), an adult male goat (two years-old) with an apron was allowed to enter in female herd at 9:00 AM and 15:00 PM for one hour each time.

**Definitions. **Goats with serum P_4_ concentration greater than or equal to 1 ng mL^-1 ^on at least two consecutive blood samplings were considered to have luteal activity.^   6 ^ Goats did not show standing heat but their serum P_4_ concentrations were greater than or equal to 1 ng mL^-1 ^were considered to silent heat. The resumption of luteal activity was classified base on serum P_4_ concentration: A) normal luteal activity if the first P_4_ rise occurred between 15 to 21 days; B) short luteal phase: more than 1 ng mL^-1 ^serum P_4_ level for less than 15 days; C) anestrus: less than 1 ng mL^-1^ serum P_4_ level during the study.

**Study 2: Detection of anestrous goats. **Thirteen non-pregnant female Abadeh ecotype goats were selected by ultrasonography and P_4_ measurement from the goat herd. The experiment was performed in winter, transitional period from the natural breeding season to anoestrus (February to March). The anestrous phase was confirmed by P_4_ measurement. Twenty and 10 days before the kisspeptin injection, blood samples were taken by jugular venipuncture. The serum was separated by centrifugation (for 10 min at 3000 *g*) and stored at –22 ˚C until assayed. Serum P_4_ concentration were measured using the same kit as the first study. Ten (10/13) goats were detected anestrus and five (5/10) female goats (mean ± SD body weight, 34.6 ± 6.3 kg) showing levels of P_4_ lower than 1.0 ng mL^-1 ^on both samplings, were selected as anestrous goats.^   34 ^

**Table 1:** Literature review of the effect of different types of Kisspeptin administration on the LH release in mammalian species

**Species**	**Sex**	**Reproduction status**	**Peptide type**	**Route** *	**Dose**	**Reference**
Human	Male	Adult	Kisspeptin-54	IV	4 pmol kg^-1^ per min, 90-min	^27^
Human	Male	Adult	Kisspeptin-10	IV	0.01-3.0 μg kg^-1^	^46^
Human	Female	Menstrual cycle	Kisspeptin-54	SC	0.4 nmol kg^-1^	^28^
Human	Female	Hypothalamic amenorrhea	Kisspeptin-54	SC	6.4 nmol kg^-1^ twice daily for 2 week	^4^ ^7^
Mouse	Male	Adult	Kisspeptin-10 Kisspeptin-54	ICV	1 fmol	^15^
Mouse	Male	Adult	Mouse kisspeptin 105-119	IP	100 μl of 10 μM	^19^
Rat	Male and female	Adult	Rodent kisspeptin-10	ICV	1 nmol	^4^ ^8^
Rat	Male	Adult	Kisspeptin-10	ICV/Peripheral	3 nmol	^49^
Rat	Male	Adult	Kisspeptin-54	ICV	1, 10 or 100 pmol	^50^
Rat	Male	Adult	Rat kisspeptin-52	ICV	0.1 nmol	^51^
Rat	Male	Adult	Rat kisspeptin-10 Human kisspeptin-10	ICV	1 nmol	^51^
Rat	Male	Adult	Rat kisspeptin-52	IV	10 nmol kg^-1^	^51^
Rat	Male	Adult	Kisspeptin-10	IV	0.3 nmol kg^-1^	^24^
Rat	Male Female	Pre-puberty	Metastin	SC	100 nmol kg^-1^ (male) 6.7 nmol (female)	^52^
Rat	Female	Cyclic, pregnant and lactating	Kisspeptin-10	ICV	0.1 pmol - 1 nmol	^53^
Monkey	Male	Agonadal juvenile	Human kisspeptin 45-54		10 μg	^45^
Monkey	Male	Adult	Human kisspeptin 45-54	IV	200 or 400 μg per hr	^54^
Monkey	Male	Agonadal juvenile	Kisspeptin-10	ICV/IV	30 μg or 100 μg	^26^
Pig	Male and female	Pre-puberty	Murine kisspeptin	ICV/Peripheral	10 or 100 μg 1, 2.5, or 5 mg	^25^
Sheep	Female	Estradiol-treated ovariectomized	Human kisspeptin 112-121	ICV	0.2 μg per min for 4 hr	19
Sheep	Female	Estradiol-treated ovariectomized	Human kisspeptin-1	IV	6 nmol	^21^
Sheep	Female	Anestrous season	Murine kisspeptin-10	IV	12.4 nmol per hr, for 30 or 48 hr	^21^
Sheep	Female	Breeding season	Murine kisspeptin-10	IV	0.48 μmol per hr over 8 hr	^21^
Sheep	Female	Anestrous season	Kisspeptin-10	IV	15.2 nmol per hr	^43^
Goat	Male	Adult	Kisspeptin-10	IV	5 µg kg^-1^	^42^
Goat	Female	Luteal phase	Kisspeptin-10	IV	1, 5 and 10 μg kg^-1^	^41^
Welsh pony	Female	Cycling	Human kisspeptin-10	IV	10 mg	^55^
Cow	Female	Pre-puberty	Kisspeptin-10	IV	1 mg	^22^
Cow	Male and female	Pre-puberty	Kisspeptin-10	IV or IM	5 μg kg^-1^	^56^
Cow	Female	Cycling	Kisspeptin-10	IV	100 or 200 pmol kg^-1^	^23^

SC: Subcutaneous; IV: Intravenous; IP: Intraperitoneal; IM: Intramuscular.

* ICV: Intracerebroventricular;

**LH-releasing responses to a single IV injection of kisspeptin. **Five goats were given a single IV injection of saline (2 mL) as control group and 1 hr apart from the later sampling of treatment group (1 μg kg^-1^, supplied by Clarke IJ, Monash University, Melbourne, Australia) as treatment group. The freely moving goats were sampled (2.5 mL blood each) or injected via an indwelling catheter previously inserted into one of the jugular veins. The blood samples were collected at –60, –40, –20, 0 (just before injection), 10, 20, 30, 40, 50, 60, 80, 100, 120 and 140 min after the injection. The goats were firstly sampled 200 min as control group and after 1 hr interval were sampled as treatment group. The serum was separated by centrifugation (for 10 min at 3000 *g*) and stored at –22 ˚C until assayed. Serum LH concentration were measured in this experiment using validated commercial ELISA kit (goat LH ELISA kit, Cusabio Biotech Co. Ltd., Wuhan, China). The analytical sensitivity of the test was typically less than 0.24 mIU mL^-1^.

**Statistical analysis. **All data from the experiments are presented as the mean ± SEM. The statistical significance of differences in serum LH concentration between the saline-treated control and kisspeptin-treated groups was analyzed by independent sample *t*-test. The statistical significance of differences in serum LH concentration between consecutive sampling times of each group was determined using paired sample *t*-test. All data were analyzed using the SPSS (Version 11.5, SPSS Inc., Chicago, USA). Results were considered significant at the *p *< 0.05 level.

## Results

**Fig. 1:**
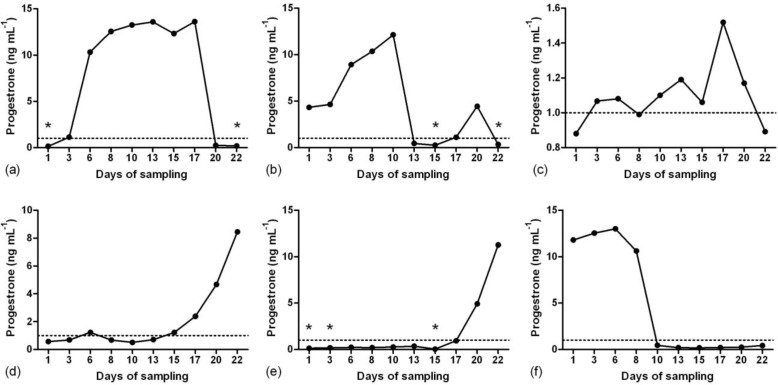
Patterns of serum progesterone concentration in six Abadeh ecotype goats in summer (non-breeding season). Samplings were done an hour after the presence of teaser buck. **a)** normal luteal activity; **b)** normal luteal activity and short luteal phase; **c)** short luteal phase and normal luteal activity; **d** and **e)** end of anestrus and beginning of luteal activity and **f)** end of luteal activity and beginning of anestrus. Stars show the time of heat detection.

**Fig. 2:**
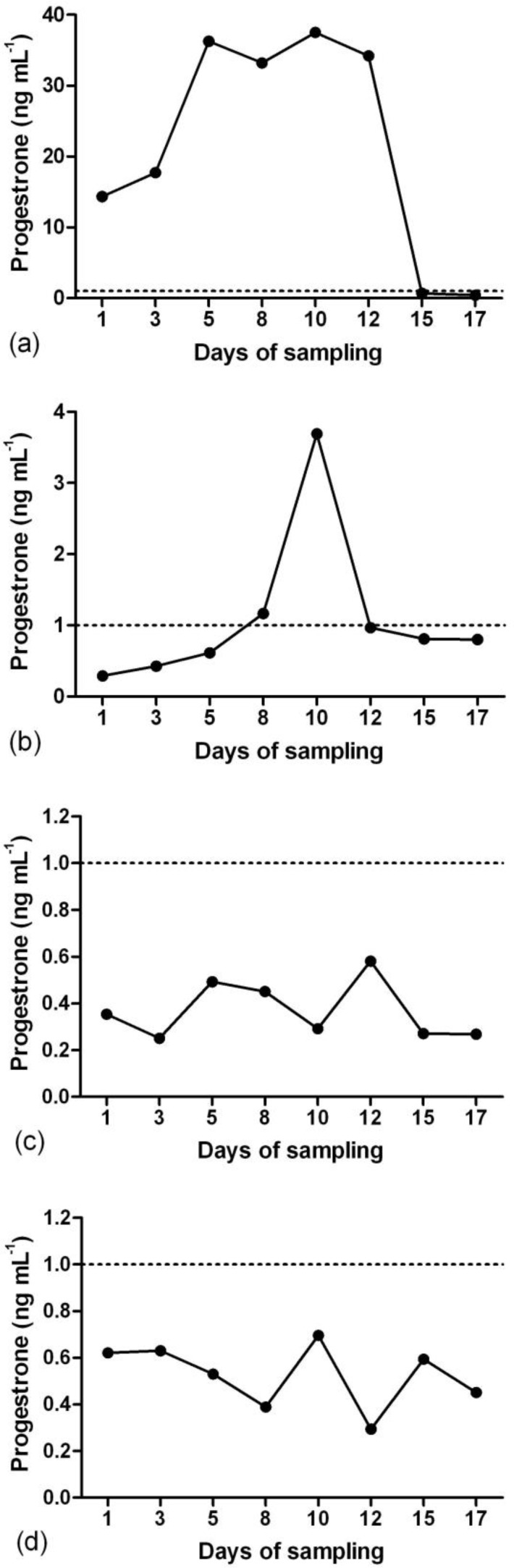
Patterns of serum progesterone concentration in four Abadeh ecotype goats in winter (breeding season) in the absence of teaser buck during the sampling days. **a)** Normal luteal activity; **b)** short luteal phase; **c** and **d)** anestrus

**Study 1. **Serum P_4_ profiles showed that during the summer in the first week of sampling, four does had luteal activity and two does were anestrus (Fig. 1). From the third week, luteal activity of two anestrous does started and one of the cyclic animals became anestrus. During the summer only three does showed estrous behavior. During the winter based on P_4_ level, two does had luteal activity and two does were anestrus (Fig. 2).

**Fig. 3:**
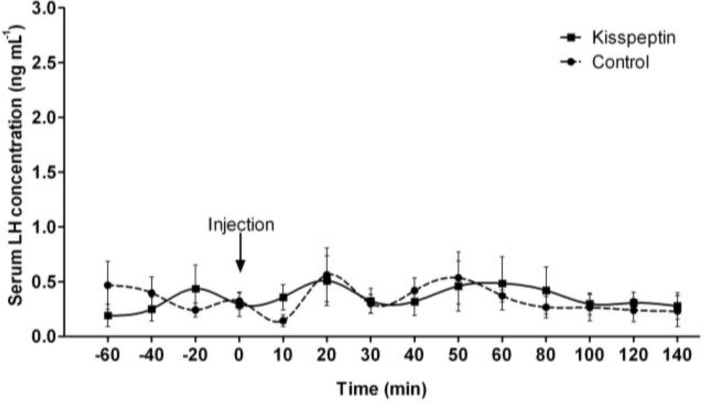
The serum luteinizing hormone concentration (LH, ng mL^-^^1^) in response to intravenous injections of 1 μg kg^-1^ body weight of kisspeptin (treatment) or saline (control) in anestrous does (Abadeh ecotype goats). Arrow indicates the time of injection. Each value represents the mean ± SEM for five animals

**Study 2. **Serum LH levels in response to a single IV injection of 1 μg kg^-1^ of kisspeptin in goats are shown in Figure 3. The level of serum LH concentration in the saline-injected control and kisspeptin treatment group in all sampling times were not significantly different (*p *> 0.05).

## Discussion

Breeding season in goats starts from late in summer.^3^^,^^5^^,^^6^ In general, summer consider as non-breeding season for animals that have five month gestation period (sheep, goat, and deer).^   35 ^ Nevertheless, based on present findings during the long days, all of six Abadeh ecotype does had luteal activity and three of them showed standing heat. In summer, two does showed luteal activity after anestrous period. Two possibilities can be raised about this finding. First, these animals entered to the breeding season normally; because it has reported that serum P_4_ concentration increased in Damascus goats during the august and breeding season starts from this month.^   36 ^ Second, since we used buck for heat detection, male effect could facilitate ovaries activation because in Cashmere anestrous does, buck could induce ovulation.^   37 ^ The “c” goat (Fig. 1) did not show standing heat or significant increase of P_4_ concentration, but pattern of P_4_ profile in this goat was more similar to “d” and “e” goats before activation of luteal activity. Silent heat in the beginning of breeding season was reported in Abadeh, Markhoz and Nubian goats.^4^^,^^5^^,^^38^ Therefore, it seems that this goat was in transition period from anestrus to breeding season.

Unlike our finding, Emady *et al*. reported that non-breeding season in native Abadeh goats last from April to August.^   4 ^ Although, they used buck for heat detection, they did not observe estrous does during the non-breeding season.^   4 ^ In the present study, estrous does had P_4_ level less than 0.4 ng mL^-1^ and shows that corpus luteum is the main source for P_4_ secretion.^   39 ^ Different P_4_ levels that we observed during the luteal phases may be depend on number and activity of corpora lutea as reported before.^40^ In spite of many goat breeds that have maximum reproductive activity during the autumn and winter,^5^^,^^6^ our finding in Abadeh ecotype goats showed that long-term maintenance does without buck cause anestrus in some animals during the short days.

Therefore, based on our finding, it can be concluded that presence of buck in the herd during the beginning of the breeding season or absence of buck in the herd during the late in breeding season lead to activation or deactivation of ovaries, respectively. Along with shortening of the day light, melatonin secretion increases and stimulates reproductive axis. However, melatonin is only one of the various factors that affect breeding season; according that, we observed anestrous animals during short-days when melatonin level is high. There are probably male effect and pheromone secretion, activate amygdale neurons and these neurons activate reproductive axis. In addition, male effect and melatonin together might stimulate GnRH neurons. The same reasoning could be true for anestrous season. In summer, long day light and absence of male could inhibit estrous cycle. Finally, it should be noted that for estrous behavior different factors such as genetic, temperature and nutrition are important and need more investigation.

This study is the first to examine the effect of kisspeptin on the secretion of LH in female anestrous goats. Our experiments demonstrated that single dose IV injections of 1 μg kg^-1 ^of kisspeptin did not induce a LH-releasing effect during anestrous season in female goats. In contrast with our study, in the luteal phase of female goats, a single injection of kisspeptin-10 (IV, 1, 5 and 10 μg kg^-1^) stimulated the release of LH.^   41 ^ Maximum values (1.5 ng mL^-1^) were observed 20 to 30 min after the injection.^   41 ^ Moreover in male goats, a single injection of kisspeptin-10 (IV, 5 µg kg^-1^) significantly stimulated the release of LH.^   42 ^ Therefore, the minimum dose level of kisspeptin-10 could not increase LH in acyclic goats.

In ovariectomized ewes, injection of ovine kisspeptin (IV, 3 mg) increased serum LH concentration. ^20^ This dose was about 10-times more than the doses used in the present study. In addition, in anestrous season, administration of human kisspeptin 112-121 (ICV, 0.2 μg per min for 4 hr) in ovariectomized estradiol treated sheep increased serum LH concentration.^   19 ^ Apart from the injection site, resulting in faster drug reaching the hypothalamus in that study, longer duration of administration compared with the present study could stimulate LH secretion in anestrous phase. On the other hand, in estradiol-treated ovariectomized ewes during the anestrous season, injections (IV, 6 nmol) of doses as low as human C- terminal decapeptide Kiss-1 elevated plasma LH in anestrous season.^   21 ^ Furthermore, infusion of kisspeptin (IV, 12.4 nmol per hr, for either 30 or 48 hr) caused ovulation in more than 80% of kisspeptin-treated ewes, whereas less than 20% of control ewes ovulated.^   21 ^ During the breeding season and in P4-synchronized cyclical ewes, constant infusion of murine C-terminal kisspeptin decapeptide-10 (IV, 0.48 μmol per hr over 8 hr) was administered 30 hr after withdrawal of a P_4_ priming period, and surge responses in LH occurred within 2 hr.^   21 ^

Murine C-terminal kisspeptin decapeptide was equi-potent to human C-terminal kisspeptin decapeptide in terms of the release of LH.^   21 ^ Infusion of kisspeptin-10 (IV, 15.2 nmol per hr) induced a well-synchronized LH surge (around 22 hr after the start of the kisspeptin infusion) in seasonally acyclic ewes.^   42 ^ Therefore, in anestrous animals, site of injections, pretreatment with estradiol or increase of kisspeptin doses or duration of administration could increase the chance of LH release in anestrous animal.

Consistent with our findings, subcutaneous injection of the 0.1, 0.3, 1 and 50 nmol kisspeptin-10 and kisspeptin-14, did not increased plasma LH at 60 min post-injection in adult male rat.^   44 ^ Moreover, a single bolus of human metastin 45-54 (IV, 10 μg) during the last 3 hr of the continuous 4 days administration of human metastin 45 to 54 in agonadal juvenile male monkeys on day 4 did not robust LH release.^   45 ^

In summary, our study presented the first effort to evaluate the influence of kisspeptin on the secretion of LH, in female anestrous goats. The results clearly showed that single dose IV injection of 1 µg kg^-1^ of kisspeptin-10 did not stimulate the release of LH during the anestrous animal in female Abadeh ecotype goats.
